# Behaviorally informed digital campaigns and their association with social media engagement and COVID-19 vaccine uptake in Belize

**DOI:** 10.1186/s44263-024-00079-w

**Published:** 2024-10-24

**Authors:** Giuliana Daga, Lajos Kossuth, Cynthia Boruchowicz, Florencia Lopez Boo, Natalia Largaespada Beer

**Affiliations:** 1 Inter-American Development Bank, Social Protection and Health Division, 1300 New York Avenue NW, Washington, DC USA; 2 Sloan School of Management, Massachusetts Institute of Technology, 100 Main St, Cambridge, MA USA; 3 Ministry of Health and Wellness, 762G+6W3, East Block Building, Independence Plaza, Belmopan, Belize

**Keywords:** COVID-19, Vaccines, Behavioral economics, Vaccine hesitancy, A/B Testing, Latin America

## Abstract

**Background:**

Increasing vaccination coverage was key to curbing the COVID-19 pandemic globally. However, lack of trust in the vaccine and fear of side effects in regions like the Caribbean resulted in a low uptake despite enough vaccine supply.

**Methods:**

We conducted two correlational analyses and one experiment between five sequential behaviorally informed Facebook campaigns, social media performance outcomes, and district-level vaccination data. First, we ran multivariate linear regression models to estimate the mean differences between the campaigns in (i) social media performance (“Clicks” and “Engagement”) and (ii) COVID-19 vaccination uptake at the district level. “Clicks” were measured by the number of people who clicked on the respective Facebook advert and visited the official vaccination site. “Engagements” were the number of people interacting with the advert through likes and emojis. Second, we took advantage of the experimental design during one of the campaigns to analyze the differential effect of messages conveying information about the number of people reporting vaccination side effects using words (“Few”/ “Majority) and numbers (“3 out of 100 “) on social media performance.

**Results:**

The correlational analysis showed that the number of “Clicks” and “Engagement” was similar among campaigns, except for the campaign focusing on vaccines’ effectiveness, which had 14.65 less clicks and 19.52 less engagements per advert (including controls and district-fixed effects) compared to the base “It’s safe” campaign. Vaccination rates were highest at times coinciding with campaigns focusing on vaccination safety and effectiveness. Our experimental results showed that informational messages related to side effects that were framed using words (“Majority did not report discomfort”/ “Few persons reported discomfort”) were better at generating “Clicks” compared to those using numbers (“3 out of 100 reported discomforts”).

**Conclusions:**

Facebook adverts highlighting vaccine safety had a similar level of social media performance as other campaigns, except for adverts focusing on vaccine efficacy, which performed worse. Communicating side-effect information with words instead of numbers can expand social media interest in low-uptake regions like the Caribbean. Our results serve as preliminary evidence for public health officials to encourage vaccine uptake in high-hesitancy contexts.

**Supplementary Information:**

The online version contains supplementary material available at 10.1186/s44263-024-00079-w.

## Background

Increasing COVID-19 vaccine uptake has been key to curbing the coronavirus pandemic. Achieving global immunity would not only protect individuals from serious diseases, hospitalizations, and deaths but also strengthen the health system, fully restart economies, and lower the risk of new and more dangerous variants [1]. That is why the World Health Organization (WHO) established the goal to inoculate 40% of the world’s population against COVID-19 in 2021 and 70% by 2022 [1, 2].

Vaccination coverage across the Caribbean was low at the beginning of 2022: by February, ten countries had yet to reach the WHO’s 2021 goal of 40% full vaccination population coverage [3]. Belize was one of the countries facing such a challenge. Although limited vaccine supply was a reason for low vaccination rates at the beginning of the pandemic, this was no longer the case in 2022. By February 2022, the country had a supply of COVID-19 vaccines for 110% of its eligible population, but only 58.7% of the country’s total population had received at least one dose [4].

Given this reality, the Belizean Ministry of Health and Wellness (MOHW) partnered with the Inter-American Development Bank (IDB) to understand the barriers to vaccination faced by its population and agree on ways to tackle them. The literature suggests that a mix of inaccessibility, low information, and sociocultural rejection issues can explain COVID-19 vaccine hesitancy [5]. To assess the importance of these factors, the MOHW and the IDB conducted quantitative surveys in October 2021 using the Integrative Model of Behavioral Prediction [6]. The Integrative Model of Behavioral Prediction is a conceptual framework that can identify which variables are the most relevant to determine a given behavior in any given population, and it proposes that a health message should address those critical determinants to improve the recommended behavior [7]. The study found that a fear of side effects related to the vaccine and a lack of trust in the vaccine were stated by the unvaccinated as the main reasons for not getting the vaccine [6]. Additionally, the study suggested that a higher perception of the vaccine’s accessibility and safety was associated with a higher likelihood of being vaccinated [6]. In February 2022, a series of focus groups were also implemented with local vaccination teams, senior citizens, parents of adolescents, and low-income and low-education groups to further understand the reasons for hesitancy among the unvaccinated.

The literature suggests that interventions narrowing the intention-action gap are among the most effective to increase vaccination. For example, in many contexts, facilitating action by sending reminders and planning prompts, facilitating logistics through the use of online booking, and providing incentives, sanctions, and requirements has proven effective, especially for citizens whose previous intentions were favorable (see, for instance, [8–10]). We also know supply-side issues and logistic systems are important for vaccination uptake [11, 12]. However, evidence showing the effectiveness of interventions to close the intention-action gap was relatively scarcer during the COVID-19 pandemic and was typically focused on developed countries (see [13–16]). The distinction of contexts (i.e., Latin America and the Caribbean vs. the USA or Europe) was particularly important as vaccine availability varied widely in the first half of 2022 [17].

Although increasing vaccine supply and accessibility is a necessary condition, affecting intentions needs a more nuanced and thorough approach. There is less evidence on the effectiveness of strategies to change people’s perceptions, intentions, beliefs, and engagement with information and the vaccination process. For instance, Dai et al. [15], in a large-scale randomized controlled trial (RCT), showed that a low-cost intervention in the USA based on short message service (SMS) reminders significantly increased the probability of COVID-19 vaccination. However, the campaign designed to affect misperceptions and beliefs about vaccination was less successful. Likewise, mass media Facebook campaigns in the USA and France trying to promote vaccination through health professionals’ testimonies and an engaging strategy aimed at activating social networks did not change citizens’ vaccination decisions [16].

Designing and evaluating efficient means of communicating vital and sensible information to affect vaccination perceptions and intentions is key. Theory and empirical evidence seem to suggest that framing messages positively (instead of negatively) is better for encouraging healthy behaviors, as they persuade people to lower sugar intake [18] or to engage in preventive health behaviors [19, 20]. In the COVID-19 pandemic context, gains-framed health messages are believed to have been more effective at increasing self-care behaviors and motivating them in others [21], while loss-framings have been shown to increase anxiety without any changes in behavior [22]. For example, framing side effects information positively instead of negatively increased vaccine intention in Australia [23].

Even though vaccine effectiveness and side effects seem to be the main concerns among the unvaccinated, finding a correct path to address them seems challenging. Two experiments conducted in 2021 in the UK showed that transparency about the effectiveness of vaccines did not impact beliefs on the efficacy of vaccines, concerns over side effects, or intentions to receive a vaccine [24]. Likewise, a study in the Netherlands found that providing a facts box (including detailed numeric information on COVID-19 vaccination benefits and harms) did not affect participants’ link clicking, compared to a generic text [25]. Finally, regarding ways to convey numerical and verbal probabilities, researchers have found high variability of interpretation depending on the context [26, 27], and that the use of numbers results in a higher likelihood of medication use at the patient-individual level in advanced economies [28]. However, we have not found studies testing this or related hypotheses in a mass-media campaign.

With the previous in mind, a multidisciplinary team from the MOHW, the IDB, and digital marketing company Idealab Studios (located in Belize City, Belize) designed and launched five behaviorally informed Facebook campaigns with the objective of closing the intention-action gap and increasing vaccination. For this paper, we first studied the correlation between those campaigns and two outcomes for social media performance: “Engagements” and “Clicks.” We believe such measures to be a precondition to increasing vaccination. “Engagement” (any action someone takes on a Facebook post, like reactions (likes, emojis, etc.) or comments) indicated that people were reacting to the message. “Clicks” were indicators of successfully raising interest in vaccination as people clicked to find a vaccination site. We further studied the correlation between the campaigns and an aggregate measure of vaccination uptake at the district level. Finally, we took advantage of an experiment conducted during one of the campaigns to analyze the effect of being exposed to three different types of messages regarding the number of people reporting vaccination side effects on the two outcomes of social media performance. To the best of our knowledge, this was one of the first attempts at linking behaviorally informed Facebook campaigns to social media performance and COVID-19 vaccine uptake in the Caribbean.

## Methods

### Intervention design

Based on the insights from surveys and focus groups, a diverse team comprising four government officials, a mass media consulting firm, and six health and behavioral economics specialists from the IDB collaborated to design five behaviorally informed Facebook campaigns. These campaigns were sequentially displayed on Belizean Facebook users’ homepages between February and June 2022, each lasting an average of 3.8 weeks. All campaigns included the sentence:*“*Click here to find your nearest vaccination site,” a phone number, and website information at the end to minimize the hassle factor and diminish the intention-to-action gap within the vaccination process.

The first campaign (“It’s safe”) stated: “It’s safe. Hundreds of thousands of Belizeans have taken the vaccine and are protected. What are you waiting for?” Its focus was on “safety,” given that this was one of the main concerns expressed by Belizeans [6]. The campaign also attempted to induce awareness through social norms, which has been found to be a significant driver for public health behaviors and for vaccination specifically [29]. This campaign included the image of a well-known nurse and the MOHW logo, given the high level of trust in public health authorities in the country found in the previous exploratory research and in other low- and middle-income settings [30].

The second campaign (“It’s effective”) expressed the following message: “It’s effective. Once vaccinated, you are less likely to catch COVID, less likely to be hospitalized, and less likely to die. What are you waiting for?” The framing of this campaign was also based on insights provided in previous research, which found that efficacy beliefs are an important factor for hesitancy among the unvaccinated [6]. This campaign aimed to emphasize the effectiveness of the COVID-19 vaccine in preventing hospitalizations and deaths. Even though the vaccine’s effectiveness was among the main concerns in Belize and worldwide [31], there is less evidence about how to address it effectively.

The third campaign (“When vaccinated…”) had an A/B testing setting, where a control version is tested against a variant version to measure which one is most successful. Three different versions of the campaign were displayed randomly among Facebook users at the same time. In particular, the campaign stated: “When vaccinated… (i) only 3 out of 100 reported discomforts”; (ii) “few people have discomfort”; or (iii) “the majority didn’t have discomfort.” The campaign’s general framing was grounded in the fear related to the side effects of the vaccine, given that these were a primary hesitancy factor in low and middle-income countries [30]. Further, the different versions of the message were aimed at testing the null hypothesis of no difference in Facebook engagement between messages about side-effects being framed using words (“few people…,” “the majority…”) and using numbers (“3 out of 100…”). In all versions of the message, the image included a picture of a nurse, given that they are among the most trusted experts in Belize [6].

The fourth campaign (“Are you Protected?”) stated: “Are you protected? Vaccination helps protect you and your family.” The wording and framing of this message were based on the results of the Integrative Model of Behavioral Prediction, which found that protecting people’s own health and that of their families motivates individuals to get vaccinated in Belize [6].

**Fig. 1:**
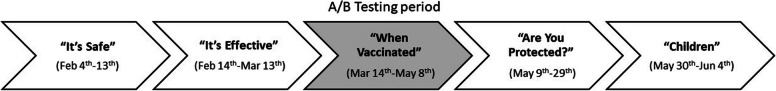
Intervention timeline (2022)

The final campaign (“Children”) expressed: “Children can now get a COVID-19 vaccine. Your child (5–11 years) can now get vaccinated at school or at your nearest vaccination site.” This campaign was informative because children were not eligible to get the COVID-19 vaccine before its launch. Figure 1 presents a timeline with a summary of the intervention.

### Data

Facebook data were collected by the Meta Ads Manager, a tool from Meta Platform, Inc. (formerly Facebook Inc.) located in Menlo Park, CA, USA. The data were then provided to us by Idealab Studios, a digital marketing company located in Belize City, Belize, founded in 2002. There were 862 unique adverts, which were our unit of observation. We defined an advert as a unique combination of campaign-time-location-district-language. There were five different campaigns comprising several adverts each. Each advert had a start and end date. They announced vaccination events in 232 different locations in Belize (e.g., public hospitals, schools, and churches), corresponding to 6 different districts. Most locations had only one advert (in English and Spanish), 62 had more than one advert, and only 17 locations had more than five adverts in each language. Adverts were displayed in English or Spanish. For each advert, Meta Ads Manager collected aggregated measures of exposure (“Reach”) and performance (i.e., the number of “Clicks” and “Engagements”) over the 230,000 Facebook users who were overall exposed to at least one of these campaigns from February to June 2022. These users made up 57.5% of Belize’s total population of approximately 400,000 people and were widely and evenly distributed across regions and towns. Additionally, 60% were between 18 and 34 and 55% were females [32]. Each advert lasted an average of 3.5 days and reached 8814 users, and generated 54 clicks (click-through rate = 0.61%) and 62 engagements (engagement rate = 0.70%—see Additional File 1: Table S1 for details).

A subset of 372 adverts belonged to the third campaign and, as such, were part of the A/B testing and were distributed in the following way: 122 observations in the first treatment arm (“only 3 out of 100 reported discomforts”), 124 in the second (“few people have discomfort,” and 126 in the third (“the majority didn’t have discomfort”). Each advert reached an average of 9106 Facebook users, lasted for an average of 3.59 days, and generated 58 clicks (click-through rate = 0.64%) and 69 engagements (engagement rate = 0.76%). Additional file 1: Table S2 shows descriptive statistics for these adverts, and Additional file 1: Table S3 shows that all three treatment arms in this study are balanced on all observed covariates.

Additionally, the MOHW provided us with official vaccination records, which contained the number of first, second, and booster shots that were applied at the district level, as reported by the vaccination teams. These data were recorded daily, and they were classified by district, vaccine brand, and type of dose. It did not include any personal identifiers. In total, the average daily first, second, and third doses during the period of study were 8.26, 11.59, and 27.15. Additional file 1: Table S4 presents summary statistics of vaccination doses. For our study, we merged these data with the Facebook data using the district and the date each advert ended.

### Empirical strategy

#### Correlational analysis: Facebook campaigns and social media performance

To assess the correlation between social media performance measured in “Clicks” and “Engagements” and each campaign, we estimated the following ordinary least squares (OLS) regression.

1 $${ y}_{h}= {\beta }_{0}+ {\beta }_{1}{\sum \text{Campaign}}_{h}+ {\beta }_{2}{\text{Reach}}_{h}+ {\beta }_{3}{X}_{h}+ {\beta }_{4}{Z}_{h}+{\gamma }_{h}+ {\varepsilon }_{h}$$

where ${y}_{h}$ was either the number of “Clicks” or “Engagements” made by groups of Belizeans exposed to Ad *h*; ${\text{Campaign}}_{h}$ were a series of dummy variables that took the value of 1 for either “It’s effective,”“When vaccinated,” “Are you protected?,” or “Children” (“It’s safe” campaign being the base group); ${\text{Reach}}_{h}$ was the number of people who were exposed to Ad *h*; ${X}_{h}$ was a vector of controls that included the daily number of new COVID-19 cases and the number of new COVID-19-related deaths at a national level on the day the advert ended, and ${Z}_{h}$ was a vector of controls that included whether the advert was run in English and the average number of days each advert ran; ${\gamma }_{h}$ were district-fixed effects; and ${\varepsilon }_{h}$ was the error term. The coefficient of interest ${\beta }_{1}$ measured the difference in the absolute number of “Clicks” and “Engagements” associated with each campaign (using the “It’s safe” campaign as a comparison).

#### Correlational analysis: Facebook campaigns and vaccination uptake

To measure the correlation between adverts and vaccination uptake on their end date, we estimated the specification from Eq. (1) again, but in this case ${y}_{h}$ was either the total number of first doses, second doses, or booster shots of the COVID-19 vaccine registered for groups of Belizeans in the district corresponding to the end date of Ad *h*. The coefficient of interest ${\beta }_{1}$ measured the difference in vaccine uptake associated with each campaign (using the “It’s safe” campaign as a comparison).

#### Experimental analysis: A/B Testing and Facebook users’ social media performance

During the “When Vaccinated” campaign, adverts were randomized into one of three differently framed messages using the Facebook A/B Testing functionality. This method, also known as a split test, is a mechanism that assigns variations of the message to the target audience to measure which one is most successful. It ensures that there are no systematic differences between groups, addressing concerns related to the previous Facebook approach that led to activity bias, targeting optimization, and competition-induced confounds [33].

We tested the null hypothesis of no differences in social media performance between adverts that include messages on vaccination side-effects probabilities using numbers vs. words, as well as positive vs. negative framing through Eq. (2).

2 $${ y}_{h}= {\beta }_{0}+ {\beta }_{1}{\sum AB}_{h}+ {\beta }_{2}{\text{Reach}}_{h}+ {\beta }_{3}{X}_{h}+{\beta }_{4}{Z}_{h}+ {\gamma }_{h}+ {\varepsilon }_{h}$$

where ${y}_{h}$ was the number of “Clicks” or “Engagements” made by groups of Belizeans exposed to Ad *h*; and ${AB}_{h}$ were dummy variables that take the value of 1 for either “few people have discomfort” or “the majority didn’t have discomfort” adverts (“only 3 out of 100 reported discomforts” being the base group). The rest of the variables were the same as in Eq. (1).

### Software implementation

We ran all linear regressions using the “regression” command in STATA MP 17. We accounted for the multiple comparisons problem in two ways. First, we re-calculated our estimated *p*-values following Romano and Wolf (2016), with 1000 bootstrap resampling iterations using the “rwolf2” command, and we calculated the Bonferroni-adjusted significance threshold manually, assuming a desired 10% significance level and eight pairwise comparisons.

## Results

### Facebook campaigns and Facebook users’ social media engagement

Table 1 shows that the “It’s safe” campaign (base category) was associated with around 14.66 more “Clicks” and 19.53 more “Engagements” when compared to the “It’s effective” campaign in the specification that included controls and district-fixed effects. Considering the 0.61% and 0.70% average click and engagement-through rate for this period, the previous results translated into a 0.17% and 0.22% higher click and engagement-through rate compared to the “It’s effective” campaign. We found no significant differences between the “When vaccinated” and the “Are you protected?” campaigns and the “It’s safe” campaigns. Finally, the “Children” campaign was, on average, associated with around 32.73 “Clicks” and 60.37 “Engagements” more than the “It’s safe” campaign. However, we should take these results with caution, given that, as shown in Additional file 1: Table S1, the number of adverts recorded during this campaign was substantially low (11), potentially leading to spurious estimations. Even though a “Click” is qualitatively a more “in-depth” relation with the Facebook advert than an “Engagement” (since users need to leave Facebook to acquire additional information), our estimations could not conclude anything related to the difference in magnitudes between the “Clicks” and “Engagements” correlational estimates. The standard errors were too big to make such assessments. Our initial estimations were robust to the multiple comparisons problem (Additional file 1: Table S5).

**Table 1:** Association of Facebook campaigns with clicks and engagements

	**Clicks**		**Engagement**	
	(i)	(ii)	(iii)	(iv)
**Campaigns**				
It’s effective	− 15.590^***^	− 14.657^***^	− 21.141^***^	− 19.527^***^
	(2.310)	(3.613)	(2.949)	(4.412)
When vaccinated	− 3.076	1.235	− 5.440^*^	− 0.004
	(2.321)	(4.140)	(2.993)	(5.063)
Are you protected?	0.973	4.173	− 6.844	− 2.735
	(3.609)	(3.900)	(4.279)	(4.594)
Children	36.492^***^	32.726^***^	64.757^***^	60.373^***^
	(7.252)	(7.433)	(9.844)	(9.801)
Reach	0.007^***^	0.006^***^	0.008^***^	0.007^***^
	(0.001)	(0.001)	(0.001)	(0.001)
Controls	NO	YES	NO	YES
District FE	NO	YES	NO	YES
Observations	862	862	862	862
*R*^2^	0.709	0.784	0.699	0.774
Adjusted *R*^2^	0.707	0.780	0.698	0.770
**Mean**	**53.503**		**62.138**	
**SD**	**57.231**		**67.499**	

^*^p < 0.1, ^**^p < 0.05, ^***^p < 0.01. OLS estimations with robust standard errors are clustered in parentheses. Columns (i) and (iii) represent our reduced form specifications. In columns (ii) and (iv), we also included a set of controls and district fixed effects. In (i) and (ii), the outcome variable of interest is the absolute number of Clicks. In (iii) and (iv), the outcome variable of interest is the absolute number of Engagements. All the coefficients associated with the Campaigns are to be interpreted with the “It’s safe” Campaign as the comparison point. Controls included the absolute number of Reaches, an indicator variable that takes the number 1 if the campaign was run in English, the number of new COVID cases, the number of new COVID-related deaths, and the average number of days each campaign ran in each location

### Facebook campaigns and vaccination uptake

The specification that included controls and district-fixed effects showed that the “It’s safe” campaign (base category) coincided with an additional uptake of 5.39 first doses, 10.04 s doses, and 21.23 booster doses compared to the “When vaccinated” campaign; an additional uptake of 5.34 first doses, 12.58 s doses, and 19.40 booster doses compared to the “Are you protected” campaign; and an additional 4.16 first doses and 13.55 s doses, compared to the “Children” campaign. To put the magnitude of these associations into perspective, the national daily average number of doses was around 8.2 for the first dose, 11.5 for the second dose, and 27.1 for the booster for the February 4th to June 4th period, respectively. Further, we found the “It’s effective” campaign to correlate with the uptake of a higher number of first doses (1.7) compared to the “It’s safe” campaign, but its significance and those related to the “Children” campaign for booster shots disappeared when adjusted for the multiple comparisons problem (Additional file 1: Table S6). A more detailed overview of these results can be found in Table 2.

**Table 2:** Associations of Facebook campaigns with vaccine uptake—first, second, and booster doses (all brands)

	**First doses**		**Second doses**		**Booster shots**	
	(i)	(ii)	(iii)	(iv)	(v)	(vi)
**Campaign**						
It’s effective	2.040^***^	1.787^**^	− 0.200	− 0.797	− 10.115^***^	− 3.140
	(0.593)	(0.820)	(0.581)	(0.681)	(2.053)	(2.276)
When vaccinated	− 5.150^***^	− 5.386^***^	− 9.464^***^	− 10.036^***^	− 30.548^***^	− 21.228^***^
	(0.548)	(0.852)	(0.455)	(0.677)	(1.925)	(2.281)
Are you protected?	− 5.178^***^	− 5.342^***^	− 12.202^***^	− 12.572^***^	− 23.491^***^	− 19.40^***^
	(0.549)	(0.678)	(0.463)	(0.533)	(1.956)	(1.960)
Children	− 3.653^***^	− 4.156^***^	− 12.306^***^	− 13.551^***^	− 7.857^***^	− 0.161
	(0.526)	(0.873)	(0.408)	(0.781)	(1.869)	(2.372)
Controls	NO	YES	NO	YES	NO	YES
District FE	NO	YES	NO	YES	NO	YES
Observations	644	644	644	644	644	644
*R*^2^	0.526	0.532	0.551	0.560	0.466	0.514
**Mean**	**8.267**		**11.599**		**27.158**	
**SD**	**4.814**		**7.241**		**14.401**	

^*^p < 0.1, ^**^p < 0.05, ^***^p < 0.01. OLS estimations with robust standard errors in parentheses. In (i) and (ii), the outcome variable of interest is the absolute number of first doses of the vaccine for all brands; in (iii) and (iv), the absolute number of second doses for all brands; and in (v) and (vi), the absolute number of booster shots for all brands. Columns (i), (iii) and (v) represent our reduced form specifications. In columns (ii), (iv), and (vi), we also included a set of controls and district fixed effects. All the coefficients associated with the campaigns are to be interpreted with the “It’s safe” campaign as a comparison. Controls included the absolute number of Reaches, an indicator variable that takes the number 1 if the campaign was run in English, the number of new COVID cases, the number of new COVID-related deaths, and the average number of days each campaign ran in each location

We performed additional estimations of (2), but this time, merging the ad’s end date with a 6-day moving average of the vaccines as our outcome variables to remove noise (see Additional file 1: Table S7). They maintained their direction and statistical significance.

### A/B Testing and Facebook users’ social media performance

Table 3 shows the results of the A/B testing. Our main finding was that messages that used the words “Few” and “Majority” generated 7.62 and 9.13 more “Clicks,” respectively, than the “3 out of a 100” framed advert in our specification with no controls. Considering the 0.64% average click-through rate for this A/B Testing period (as shown in Additional file 1: Table S2), the previous results translated into a 0.08% and 0.10% higher click-through rate for the “Few” and “Majority” framings, compared to the “3 out of 100,” respectively. In the specification including controls, we only found a slightly significant effect for the “Majority” treatment vis-a-vis “3 out of 100.” These results stood for “Clicks” but not for “Engagements” (we did not find enough statistical evidence to reject the null hypotheses for the latter). The specification without controls was robust to the multiple hypothesis problem only for “Majority” vs. “3 out of 100” at a 10% confidence level using the Romano–Wolf *p*-value but not Bonferroni, as shown in Additional file 1: Table S9.

**Table 3:** A/B Testing—change in clicks and engagements—3 out of 100 as the basis

	**Clicks**		**Engagements**	
	**(i)**	**(ii)**	**(iii)**	**(iv)**
**When vaccinated**				
Few persons	7.616^*^	7.819^**^	6.963	7.430
	(4.441)	(3.813)	(5.627)	(4.851)
Majority	9.132^**^	7.454^*^	8.643	6.563
	(4.472)	(4.087)	(5.474)	(5.021)
Reach	0.008^***^	0.008^***^	0.009^***^	0.009^***^
	(0.001)	(0.001)	(0.001)	(0.001)
Controls	NO	YES	NO	YES
District FE	NO	YES	NO	YES
Observations	372	372	372	372
*R*^2^	0.754	0.815	0.742	0.806
Adjusted *R*^2^	0.752	0.808	0.740	0.799
**Mean**	**58.393** **69.880**		**68.857** **83.614**	
**SD**				

^*^p < 0.1, ^**^p < 0.05, ^***^p < 0.01. OLS estimations with robust standard errors in parentheses. Columns (i) and (iii) represent our reduced form specifications. In columns (ii) and (iv), we also included a set of controls and district fixed effects. In (i) and (ii), the outcome variable of interest is the absolute number of Clicks. In (iii) and (iv), the outcome variable of interest is the absolute number of Engagements. All the coefficients associated with the alternatives of the “When vaccinated” Campaign are to be interpreted with the “3 out of a 100” alternative as the comparison point. Controls included the absolute number of Reaches, an indicator variable that takes the number 1 if the campaign was run in English, the number of new COVID cases, the number of new COVID-related deaths, and the average number of days each campaign ran in each location

To test for differences between the “positive” (“Majority did not report discomforts”) and “negative” (“Few reported discomforts) loads of the messages, we changed the base category in (2) from “3 out of 100” to “Majority” and re-estimated the equation. The results are presented in Table 4. We found that even though the sign for “Few Persons” is negative (compared to the “Majority” campaign), the difference is not statistically significant.

**Table 4:** A/B Testing—change in clicks and engagements—majority as the basis

	**Clicks**		**Engagements**	
	**(i)**	**(ii)**	**(iii)**	**(iv)**
**When vaccinated**				
3 out of a 100	− 9.132^**^	− 7.454^*^	− 8.643	− 6.563
	(4.472)	(4.087)	(5.474)	(5.021)
Few persons	− 1.516	0.365	− 1.679	0.867
	(4.555)	(3.947)	(5.391)	(4.635)
Reach	0.008^***^	0.008^***^	0.009^***^	0.009^***^
	(0.001)	(0.001)	(0.001)	(0.001)
Controls	NO	YES	NO	YES
District FE	NO	YES	NO	YES
Observations	372	372	372	372
*R*^2^	0.754	0.815	0.742	0.806
Adjusted *R*^2^	0.752	0.808	0.739	0.799
**Mean** **SD**	**58.393** **69.880**		**68.857** **83.614**	

^*^p < 0.1, ^**^p < 0.05, ^***^p < 0.01. OLS estimations with robust standard errors in parentheses. Columns (i) and (iii) represent our reduced form specifications. In columns (ii) and (iv), we also included a set of controls and district fixed effects. In (i) and (ii), the outcome variable of interest is the absolute number of Clicks. In (iii) and (iv), the outcome variable of interest is the absolute number of Engagements. All the coefficients associated with the alternatives of the “When vaccinated” Campaign are to be interpreted with the “Majority” alternative as the comparison point. Controls included the absolute number of Reaches, an indicator variable that takes the number 1 if the campaign was run in English, the number of new COVID cases, the number of new COVID-related deaths, and the average number of days each campaign ran in each location

## Discussion

Even though much research has focused on understanding the reasons behind hesitancy and interventions to reduce the intention-to-action gap, evidence on effective strategies to affect vaccination intention remains limited. To the best of our knowledge, this paper was the first study to quantify the importance of using social media to inform citizens about the COVID-19 vaccination process in a context of high hesitancy, like the Caribbean. The study was timely, given the campaigns were run in early 2022 when booster shots were only starting to be rolled out in Belize. Furthermore, it was among the first studies that capitalized on the Facebook A/B testing tool to experimentally test the differential impact of various messaging framings on Belizeans’ willingness to access information about the COVID-19 vaccination process.

Health communication campaigns can improve the population’s health, especially when considering behavioral theories and cognitive biases [34, 35]. While our correlational analysis results should be taken cautiously, given that the campaigns were delivered sequentially and other temporal confounders might be at play apart from our controls (i.e., the number of new COVID-19 cases and deaths), some lessons are worth highlighting.

First, we saw that all the campaigns, except for “It’s effective,” had a similar level of social media performance among Belizeans. Moreover, the “It’s safe” and “It’s effective” campaigns coincided with the highest uptake of vaccination doses and the “Children” campaign with booster doses. Our results were intriguing: even though safety and effectiveness were reported as primary concerns globally [31] and significant predictors of vaccination in Belize [6], highlighting the latter coincided with lower numbers of clicks and engagement in Belize. This might suggest that the design of the messages and other factors could be equally important in explaining vaccination interest and uptake. For example, we know that vaccine access generally contributed to low uptake in Belize and worldwide [5, 6]. The phased-in administration of vaccines in Belize could explain vaccination behavior in some contexts. Further research with experimental designs should be used to test this hypothesis.

Regarding the A/B experiment, we found that messages using words to reflect the probabilities of suffering side effects were more effective than using numbers. This aligns with previous evidence showing that providing detailed information about vaccine efficacy and side effects in communication campaigns does not influence vaccine uptake intentions [24, 36]. Although limited, previous research conducted in the health domain in advanced economies found that patients interpret verbal probabilities in a highly variable way and prefer quantitative risk information [27]. However, these studies looked at health communications at the patient-individual level rather than within a population-level public health campaign and in the context of a pandemic. Additionally, the literature suggests that positively framed messages are more effective at getting people to acquire relevant information, especially regarding health issues [20, 23]. However, even though our estimator showed a negative sign for the negative framing, we could not reject the null of no effect of this hypothesis, probably due to a lack of statistical power.

We acknowledge that providing clear and transparent information regarding what is known about the efficacy and side effects of vaccination is imperative from an ethical point of view. In this study, we analyzed the effects of social media interaction with the mass media Facebook advert, but it is important to note that very detailed epidemiological data were shared at every point in time with the public on the official MOHW Facebook website. Moreover, even though wider public health emergency communication guidelines do not tackle this issue directly, they mention that risk should not be explained in technical terms, as this is not helpful for promoting risk mitigation behaviors [37].

Our study faced some limitations. First, because of our data, we could only ascertain the causal effects of exposure to messages within the third campaign (A/B testing). Second, in all our estimations, we used advert-level aggregated mass media campaigns that further complicated the use of individual-level controls, such as vaccine beliefs and attitudes, which we know strongly influence vaccination decisions. Advert-level aggregated data could compromise the assumption of independence of observation units for OLS regressions since some locations featured more than one advert in our study’s period. Third, we did not have enough data to account for vaccine availability, which we know is an important factor for uptake. Fourth, the five behaviorally informed Facebook campaigns were displayed sequentially and not randomly to users between February and June 2022. Therefore, while the Facebook campaigns were being implemented, several activities were taking place at the same time, given that there were batches of vaccines in the country that were set to expire soon. This limited our chances of controlling all possible confounders in correlational analyses. Finally, our statistical power could have been better with larger samples.

It is important to highlight that this paper was not intended to study an experiment in isolation but rather to capture learnings and best practices of policy interventions in the most rigorous way possible given the reality constraints. The previous meant that different attempts to increase awareness and vaccine uptake were put in place simultaneously, and changes were executed after quick rounds of feedback due to the urgency. The results have practical implications for decision-makers.

## Conclusions

We found some evidence that Facebook adverts highlighting vaccine safety had a similar level of social media performance as the others, except for the adverts focusing on its efficacy. Moreover, vaccination rates were highest at times coinciding with campaigns that highlighted safety and efficacy. We additionally found proof that framing public health adverts aimed at informing about probabilities of suffering side effects was more effective using words rather than numbers (“Majority/Few persons” vis-a-vis “3 out of a 100”). However, we believe further research should be conducted to address people’s concerns with vaccination and incentivize action more effectively. Our results serve as preliminary evidence for public health officials to encourage vaccine uptake in high-hesitancy contexts.

## Supplementary Information

Additional file1: Table S1.Summary statistics of main variables: social performance measurements by Facebook campaign. Table S2. Summary statistics of social performance variables by A/B test treatment arm. Table S3. Balance table by A/B test treatment arm. Table S4. Summary statistics of main variables: daily vaccination uptake by Facebook campaign. Table S5. Family-wise error corrections: association of Facebook campaigns with clicks and engagements. Table S6. Family-wise error corrections: associations of Facebook campaigns with vaccine uptake: first, second, and booster doses (all brands). Table S7. Associations of Facebook campaigns with vaccine uptake: first, second, and booster doses (all brands – moving averages). Table S8. Family-wise error corrections –A/B testing.

## Data Availability

Anonymized and aggregated datasets are available in the IDB Social Data repository (https://scldata.iadb.org/es/public). Access can be granted upon request by emailing scldata@iadb.org. The MOHW maintains a strict policy of keeping vaccination data confidential.
