# Supplementary material for: Behaviorally informed digital campaigns and their association with social media engagement and COVID-19 vaccine uptake in Belize
Source: BMC Glob Public Health. 2024 Oct 24;2:71. doi: 10.1186/s44263-024-00079-w (PMC11622853; doi:10.1186/s44263-024-00079-w)
Supplement: Supplementary file 1 — Additional file1: Table S1.Summary statistics of main variables: social performance measurements by Facebook campaign. Table S2. Summary statistics of social performance variables by A/B test treatment arm. Table S3. Balance table by A/B test treatment arm. Table S4. Summary statistics of main variables: daily vaccination uptake by Facebook campaign. Table S5. Family-wise error corrections: association of Facebook campaigns with clicks and engagements. Table S6. Family-wise error corrections: associations of Facebook campaigns with vaccine uptake: first, second, and booster doses (all brands). Table S7. Associations of Facebook campaigns with vaccine uptake: first, second, and booster doses (all brands – moving averages). Table S8. Family-wise error corrections –A/B testing. [file 44263_2024_79_MOESM1_ESM.pdf]

Behaviorally informed digital campaigns and their association with social media engagement and COVID-19 vaccine take-up in Belize

Additional file 1

Table S1. Summary statistics of main variables – Social performance measurements by Facebook campaign

	**It´s safe…**	**It´s effective…**	**When vaccinated…**	**Are you…?**	**Children…**	**Total/ Week**
	**Mean/SD**	**Mean/SD**	**Mean/SD**	**Mean/SD**	**Mean/SD**	**Mean/ SE**
**Dates**	**Feb 4th – Feb 13th**	**Feb 14th – Mar 13th**	**Mar 14th – May 8th**	**May 9th – May 30th**	**May 31th – Jun 4th**	
Clicks	35.514	36.328	58.393	71.760	144.272	53.503
	(21.277)	(32.168)	(69.880)	(50.434)	(51.521)	(57.231)
Engagement	44.114	42.048	68.856	78.287	192.909	62.138
	(25.086)	(36.540)	(83.613)	(54.445)	(65.671)	(67.499)
Reach	5413.086	7747.245	9106.151	10431.92	15,695.730	8813.974
	(3144.432)	(5656.522)	(7457.516)	(5538.252)	(5,729.943)	(6572.869)
English	0.628	0.520	0.500	0.520	0.454	0.515
	(0.490)	(0.500)	(0.500)	(0.501)	(0.522)	(0.500)
New COVID cases	131.428	28.909	6.625	69.972	0	29.730
	(109.356)	(43.181)	(9.585)	(33.120)	0	(47.536)
New COVID deaths	0	0.713	0.437	0	0	0.431
	0	(1.759)	(1.163)	0	0	(1.301)
Days x Ad, Campaign,	2.771	3.674	3.593	3.246	5	3.546
	(0. 843)	(1.192)	(1.143)	(1.240)	(0)	(1.188)

and Location						
**N**	35	286	384	146	11	862

Table S2. Summary statistics of social performance variables – by AB test treatment arm

	3 out of a 100…	Few persons…	Majority…	Total
	Mean/SD	Mean/SD	Mean/SD	Mean/SD
Clicks	53.360<br>(58.920)	64.733<br>(76.121)	59.817<br>(75.466)	58.393<br>(69.880)
Engagement	64.385<br>(74.567)	75.814<br>(89.448)	69.849<br>(88.925)	68.856<br>(83.613)
Reach	9207.664<br>(7010.214)	9,669.185<br>(8,032.018)	8,879.119<br>(7,515.268)	9106.151<br>(7457.516)
English	0.500<br>(0.502)	0.500<br>(0.502)	0.500<br>(0.501)	0.500<br>(0.500)
New COVID cases	5.885<br>(8.647)	7.129<br>(9.793)	7.253<br>(10.450)	6.763<br>(9.662)
New COVID deaths	0.459<br>(1.172)	0.451<br>(1.164)	0.380<br>(1.123)	0.430<br>(1.150)
Days x Ad and Location	3.529<br>(1.212)	3.571<br>(1.070)	3.666<br>(1.165)	3.589<br>(1.149)
**N**	122	124	126	372

Table S3. Balance table, by AB test treatment arm

	3 out of a 100	Few Persons	Majority	t-test difference		
	Mean/SE			(1)-(2)	(1)-(3)	(2)-(3)
Reach	9207.664	9669.185	8879.119	-461.522	328.545	790.066
	[634.675]	[721.296]	[669.513]			
English	0.5	0.5	0.5	0.000	0.000	0.000
	[0.045]	[0.045]	[0.045]			
New COVID cases	5.885	7.129	7.253	-1.244	-1.369	-0.125
	[0.783]	[0.880]	[0.931]			
New COVID deaths	0.459	0.451	0.380	0.007	0.078	0.071
	[0.106]	[0.105]	[0.100]			
Days x Campaign and Location	3.529	3.571	3.666	-0.042	-0.137	-0.095
	[0.110]	[0.096]	[0.104]			

Note: * $p < 0.1$, ** $p < 0.05$, *** $p < 0.01$. This table shows means and standard errors of all the controls used in the main estimations, and t-test estimates of the differences between these means for all treatment arms compared against each other. A significant t-test estimate would suggest an unbalanced sample.

Table S4. Summary statistics of main variables – Daily vaccination uptake by Facebook campaign

	It´s safe…	It´s effective…	When vaccinated…	Are you…?	Children can now…	Total
	**Mean/SD**	**Mean/SD**	**Mean/SD**	**Mean/SD**	**Mean/SD**	**Mean/SD**
First dose	10.081	12.121	4.931	4.904	6.428	8.267
	(3.145)	(4.487)	(2.036)	(1.893)	(0)	(4.814)
Second dose	17.306	17.105	7.842	5.103	5	11.599
	(2.438)	(6.819)	(2.724)	(2.649)	(0)	(7.241)
Booster	45.428	35.314	14.880	21.937	37.571	27.158
	(11.172)	(13.992)	(6.224)	(6.968)	(0)	(14.401)
**N**	35	272	180	146	11	644

Table S5. Family-Wise Error Corrections - Association of Facebook Campaigns with Clicks and Engagements

	Model p-values	Romano-Wolf p-value	Bonferroni corrections
	(i)	(ii)	(iii)
**Clicks**			
It's effective…	0.0001	0.0020	0.0010
When vaccinated…	0.7655	0.8082	1.0000
Are you…?	0.2849	0.4625	1.0000
Children can now…	0.0000	0.0020	0.0000
**Engagements**			
It's effective…	0.0000	0.0020	0.0000
When vaccinated…	0.9993	0.9980	1.0000
Are you…?	0.5518	0.7493	1.0000
Children can now…	0.0000	0.0010	0.0000

Note: This table presents multiple hypothesis testing corrected p-values for regressions of Engagements and Clicks on the campaign indicators, taking the "It's safe" campaign as the control category. Columns (ii) and (iii) present p-values corrected for the familywise error rate, i.e. the probability of making any Type 1 error. The adjusted p-values in Column (ii) are calculated following Romano and Wolf (2016), with 1000 bootstrap resampling iterations. In Column (iii), we apply a Bonferroni correction by multiplying the original p-values by the number of hypothesis tests (8: 4 treatments and 2 outcomes) and capping the adjusted p-values at 1.000. Estimations correspond to our preferred specifications, adding covariates and dummies to the linear regressions with robust standard errors.

Table S6. Family-Wise Error Corrections - Associations of Facebook campaigns with Vaccine Uptake – First, Second, and Booster Doses (All Brands)

	Model p-values	Romano-Wolf p-value	Bonferroni corrections
**First Doses**			
It's effective…	0.0297	0.1079	0.3564
When vaccinated…	0.0000	0.0010	0.0000
Are you…?	0.0000	0.0010	0.0000
Children can now…	0.0000	0.0010	0.0000
**Second Doses**			
It's effective…	0.2423	0.3556	0.001
When vaccinated…	0.0000	0.0010	0.0000
Are you…?	0.0000	0.0010	0.0000
Children can now…	0.0000	0.0010	0.0000
**Boosters**			
It's effective…	0.1683	0.3127	1.000
When vaccinated…	0.0000	0.0010	0.0000
Are you…?	0.0000	0.0010	0.0000
Children can now…	0.9457	0.9411	1.000

Note: This table presents multiple hypothesis testing corrected p-values for regressions of Engagements and Clicks on the campaign indicators, taking the "It's safe" campaign as the control category. Columns (ii) and (iii) present p-values corrected for the familywise error rate, i.e. the probability of making any Type 1 error. The adjusted p-values in Column (ii) are calculated following Romano and Wolf (2016), with 1000 bootstrap resampling iterations. In Column (iii), we apply a Bonferroni correction by multiplying the original p-values by the number of hypothesis tests (12: 4 treatments and 3 outcomes) and capping the adjusted p-values at 1.000. Estimations correspond to our preferred specifications, adding covariates and dummies to the linear regressions with robust standard errors.

Table S7. Associations of Facebook campaigns with vaccine uptake: first, second, and booster doses (all brands – moving averages)

	First doses		Second doses		Booster shots	
	(i)	(ii)	(iii)	(iv)	(v)	(vi)
**Campaign**						
It´s effective	0.288***	-0.214	-0.132	-0.383**	-9.757***	-5.004***
	(0.108)	(0.144)	(0.167)	(0.194)	(0.730)	(1.227)
When vaccinated	-6.909***	-7.511***	-9.564***	-9.800***	-32.041***	-25.893***
	(0.122)	(0.166)	(0.156)	(0.206)	(0.559)	(1.239)
Are you protected?	-7.752***	-8.037***	-12.462***	-12.599***	-26.619***	-23.681***
	(0.110)	(0.143)	(0.093)	(0.149)	(0.668)	(1.050)
Children	-7.264***	-7.801***	-12.733***	-13.066***	-19.956***	-13.522***
	0.083)	(0.161)	(0.056)	(0.234)	(0.521)	(1.312)
Controls	NO	YES	NO	YES	NO	YES
District FE	NO	YES	NO	YES	NO	YES
Observations	644	644	644	644	644	644
R^2	0.925	0.928	0.880	0.883	0.753	0.782
Adjusted R^2	0.925	0.927	0.880	0.881	0.751	0. 778
**Mean**	**7.763**		**10.914**		**25.075**	
**SD**	**3.931**		**5.864**		**12.431**	

Note: * p < 0.1, ** p < 0.05, *** p < 0.01. OLS estimations with robust standard errors in parentheses. In (i) and (ii), the outcome variable of interest is the moving average of first doses of the vaccine for all brands, in (iii) and (iv), the moving average of second doses for all brands; and in (v) and (vi), the moving average of booster shots for all brands. Columns (i), (iii) and (v) represent our reduced form specifications. In columns (ii), (iv) and (vi) we also include a set of controls and District Fixed Effects. All the coefficients associated with the campaigns are to be interpreted with the "It´s safe" campaign as the comparison point. Controls include the absolute number of Reaches, an indicator variable that takes the number 1 if the campaign was run in English, the number of new COVID cases, the number of new COVID-related deaths, and the average number of days each campaign ran in each Location

Table S8. Family-wise error corrections – AB testing

	Model p-values	Romano-Wolf p-value	Bonferroni corrections
	(i)	(ii)	(iii)
**3 out of a 100 as basis**			
**Clicks**			
Few persons	0.0873	0.1479	0.3492
Majority	0.0418	0.0879	0.1672
**Engagements**			
Few persons	0.2167	0.1948	0.8668
Majority	0.1152	0.1868	0.4608
**Majority as a basis**			
**Clicks**			
3 out of a 100	0.0418	0.0879	0.1672
Few persons	0.7394	0.8062	1.0000
**Engagements**			
3 out of a 100	0.1152	0.2038	0.4608
Few persons	0.7555	0.8062	1.0000

Note: This table presents multiple hypothesis testing corrected p-values for regressions of Engagements and Clicks on the AB campaign indicators, taking, first, the "Majority" sub-campaign as the control category; second, we take the "3 out of a 100" sub-campaign as the control category. Columns (ii) and (iii) present p-values corrected for the familywise error rate, i.e. the probability of making any Type 1 error. The adjusted p-values in Column (ii) are calculated following Romano and Wolf (2016), with 1000 bootstrap resampling iterations. In Column (iii), we apply a Bonferroni correction by multiplying the original p-values by the number of hypothesis tests (4: 2 treatments and 2 outcomes) and capping the adjusted p-values at 1.000. Estimations correspond to our preferred specifications, adding covariates and dummies to the linear regressions with robust standard errors.
